# Commentary: Possible involvement of lysosomal dysfunction in pathological changes of the brain in aged progranulin-deficient mice

**DOI:** 10.3389/fnagi.2016.00011

**Published:** 2016-02-03

**Authors:** Timothy J. Sargeant

**Affiliations:** Lysosomal Diseases Research Unit, South Australian Health and Medical Research InstituteAdelaide, SA, Australia

**Keywords:** Alzheimer's disease (AD), Parkinson's disease, frontotemporal dementia (FTD), lysosomal storage diseases, lysosomes, neurodegenerative diseases, progranulin, glucocerebrosidase gene

The recent paper by Tanaka and colleagues details the neuropathological consequences of progranulin knock out in a mouse model. Loss of progranulin function by *GRN* mutation causes neuronal ceroid lipofuscinosis, also known as Batten's disease—a progressive neurodegenerative condition belonging to a class of disorders called lysosomal storage diseases (LSD, reviewed in Kollmann et al., 2013). The unexpected link between *GRN* and Batten's disease was discovered by genetic analysis of two siblings presenting with neurological symptoms and histopathological signs consistent with LSD (Smith et al., 2012). This unanticipated genetic relationship shows progranulin is pivotal for lysosomal function in the brain, but the mechanism behind why this protein is essential remains unclear.

Of note, Tanaka et al. (2014) show that LSD caused by progranulin deficiency in mice causes severe neurodegeneration in the somatosensory thalamus (ventroposterior medial/ventroposterior lateral thalamic nuclei—VPM/VPL). Here the authors correctly point out that this is a feature shared by other types of murine Batten's disease models (Partanen et al., 2008; Kuronen et al., 2012). However, this has also been observed in other kinds of lysosomal storage disorders and is not specific to the type of storage product found in the lysosome. The lipid-storing LSD Niemann Pick disease type C1 (cholesterol, ganglioside storage), Sandhoff disease (GM2 ganglioside storage), and Gaucher disease (glucosylceramide storage) all show the same pattern of neuronal cell death in the VPM/VPL (Yamada et al., 2001; Farfel-Becker et al., 2011; Sargeant et al., 2011). Why these thalamic nuclei show selective vulnerability to diverse causes of lysosomal disorder remains unclear. It is reasonable to conclude, however, that the cause of cell death probably stems from generic lysosomal dysfunction, not from a build-up of a hypothetical disease-specific metabolite.

Having shown loss of progranulin precipitates pathological features similar to other diverse LSD, the authors of this study discuss the relevance of progranulin haplo-insufficiency to later onset neurodegenerative disease. Heterozygous mutations in the progranulin gene, *GRN*, cause familial frontotemporal lobar dementia (FTD). This was discovered when Baker et al. (2006) identified mutations in *GRN* as risk factors for FTD at 17q21, in addition to *MAPT*, the gene that encodes tau. It is likely that *GRN* is haplo-insufficient, revealed by heterozygous null *GRN* mutations in FTD. Remarkably, genetic variation in *GRN* has also been linked to other late onset neurodegenerative diseases such as Alzheimer's disease (Perry et al., 2013).

There is, however, a more fundamental process that links *GRN* mutation with late onset disease. It is now clear that progranulin is a bone fide lysosomal protein that is vital for efficient lysosomal function. This is evidenced by its obvious lysosomal subcellular localization (Gowrishankar et al., 2015) as well as its role in LSD. In light of these findings, it is important to extend the discussion presented by Tanaka and colleagues to include parallels between *GRN* and *GBA*.

Like *GRN, GBA* encodes a lysosomal protein (glucocerebrosidase, GCase). Homozygous or compound heterozygous loss of function mutations in this gene cause a lysosomal disorder called Gaucher disease. Like Batten's disease caused by *Grn* knockout in mice, *Gba* knockout produces similar neuropathology; both mouse models display conspicuous loss of neurons in the VPM/VPL that is accompanied by marked gliosis (Farfel-Becker et al., 2011; Tanaka et al., 2014). The similarities do not end there. Heterozygous mutations in both of these lysosomal genes are risk factors for late onset neurodegenerative disease. Heterozygous mutation of *GBA* is over-represented in Parkinson's disease patients (Gan-Or et al., 2008). This was initially uncovered by astute clinical observation (Rogaeva and Hardy, 2008) and the association between genetic variation in *GBA* and Parkinson's disease has since been corroborated by numerous genetic studies.

The obvious parallels between *GBA* and *GRN* haplo-insufficiency raise immediate questions about the nature of pathogenesis in later onset neurodegenerative diseases such as FTD, Alzheimer's disease and Parkinson's disease. Is risk for late onset neurodegenerative disease from mutation in *GBA* or *GRN* related to gene-product specific mechanisms? Or is it general inefficiency in lysosomal flux that causes or contributes to late onset neurodegenerative disease? Surely, the fact that mutations in two very different LSD causing genes that also contribute to late onset neurodegenerative disease suggests variation in lysosomal flux is important. This idea has been further corroborated by a study that showed FTD associated with mutation in the lysosomal network gene, *CHMP2B*, was accompanied by neuronal lysosomal storage material (Clayton et al., 2015).

With this in mind, are mutations in other lysosomal proteins risk factors for late onset neurodegenerative disorders? With over 50 genes that cause lysosomal storage disease (Cox and Cachón-González, 2012) and at least 900 genes involved in maintaining efficient lysosomal network flux (Di Fruscio et al., 2015), there are many potential candidates for genetic contribution to sporadic forms of late onset neurodegenerative disease. Future studies should address key questions presented in this commentary by focussing on the measurement and enhancement of lysosomal flux in late onset neurodegenerative disease.

In conclusion, the study by Tanaka and colleagues directly links homozygous loss of progranulin to other models of LSD. Lysosomal storage of un-degraded material, along with regionally specific neuronal cell death consistent with other diverse lysosomal disease models is strong evidence that progranulin deficiency causes lysosomal storage disease. Further to this, the role of heterozygous *GRN* mutation in late onset neurodegenerative disorders appears similar to the relationship between *GBA*, another lysosomal gene, and Parkinson's disease. This suggests generalized reduction in lysosomal network flux may be a key driver of pathogenesis in late onset neurodegenerative disease.

## Author contributions

The author confirms being the sole contributor of this work and approved it for publication.

### Conflict of interest statement

The author declares that the research was conducted in the absence of any commercial or financial relationships that could be construed as a potential conflict of interest.
